# Comparative Proteomic Analysis of Lipoprotein(a): Method-Dependent Profiles and Disease Pathways

**DOI:** 10.3390/jcm15072559

**Published:** 2026-03-27

**Authors:** Nelsa Matienzo, Zoe Kress, Sasha A. Singh, Masanori Aikawa, Rajesh K. Soni, Yihao Li, Gissette Reyes-Soffer

**Affiliations:** 1 Division of Preventive Medicine and Nutrition, Department of Medicine, Columbia University Vagelos College of Physicians and Surgeons (P&S), 630 West 168th Street, New York, NY 10032, USA; 2 Center for Interdisciplinary Cardiovascular Sciences, Division of Cardiovascular Medicine, Department of Medicine, Brigham Women’s Hospital, Harvard Medical School, Boston, MA 02115, USA; 3 Center for Excellence in Vascular Biology, Division of Cardiovascular Medicine, Brigham and Women’s Hospital, Harvard Medical School, Boston, MA 02115, USA; 4 Channing Division of Network Medicine, Department of Medicine, Brigham Women’s Hospital, Harvard Medical School, Boston, MA 02115, USA; 5 Proteomics and Macromolecular Crystallography Shared Resource, Herbert Irving Comprehensive Cancer Center, Columbia University, New York, NY 10032, USA

**Keywords:** lipoprotein(a), cardiovascular disease, Lp(a) proteome, proteomics, in-solution proteolysis, in-gel digestion

## Abstract

**Background**: Lipoprotein(a) [Lp(a)] is a genetically determined risk factor for atherosclerotic cardiovascular disease (ASCVD). Proteomic studies suggest that Lp(a)-associated proteins mediate inflammation, thrombosis, and vascular calcification, but methodological variability may influence proteome definition. **Methods**: Lp(a) was immunoprecipitated from human plasma using an apo(a)-specific monoclonal antibody and analyzed by mass spectrometry following either in-gel digestion or automated in-solution proteolysis. Proteins identified by ≥3 unique peptides and consistently detected across all samples by both methods were considered high confidence. Functional enrichment and interaction networks were assessed using STRING. **Results**: In-solution proteolysis identified 92 proteins and in-gel digestion identified 55 proteins, with 34 proteins shared between methods. These high-confidence proteins were enriched for pathways involved in lipoprotein remodeling, coagulation regulation, vesicle-mediated transport, lipid binding, and extracellular matrix organization, providing biological insight into mechanisms linking Lp(a) to inflammation, thrombosis, and calcification. **Conclusions**: Proteome composition of Lp(a) is method-dependent; however, a rigorously defined core proteome of 34 proteins was consistently identified across analytical approaches, highlighting biologically relevant pathways that may underlie Lp(a)-mediated ASCVD risk.

## 1. Introduction

**Figure 1:**
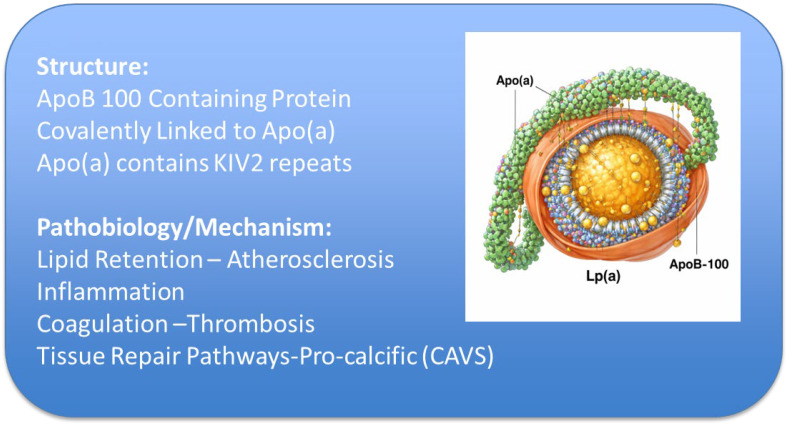
Representation of Lp(a) structure and its role in cardiovascular disease. Lp(a) is an apoB-100-containing particle linked to apo(a). Apo(a) is highly polymorphic glycoprotein with kringle domains. The particle contains cholesteryl esters and phospholipids, organized around apoB-100, with apo(a) covalently attached via a disulfide bond. The particle is associated with multiple pathogenic pathways. ApoB mediates lipid deposition and atherogenesis, while apo(a) is implicated in pro-inflammatory and pro-thrombotic processes. Lp(a)-associated proteins further contribute to inflammation, coagulation, and tissue remodeling. Disease-specific alterations in the Lp(a) proteome, including enrichment in pro-calcific factors, may underlie its role in conditions such as atherosclerosis and calcific aortic valve disease.

Lipoprotein(a) [Lp(a)] is a genetically determined and independent causal risk factor for atherosclerotic cardiovascular disease (ASCVD) [1]. Lp(a) is composed of an apolipoprotein B-100 (apoB)-containing low-density lipoprotein (LDL) particle covalently linked to apolipoprotein(a) [apo(a)] (Figure 1) [2]. ApoB-100, synthesized in the liver, serves as the structural scaffold of atherogenic lipoproteins, including very-low-density lipoprotein (VLDL), intermediate-density lipoprotein (IDL), and LDL, and directly promotes arterial lipid retention, atherogenesis, and plaque instability [3].

Emerging proteomic and functional data indicate that the composition and metabolic context of Lp(a) are dynamically modulated in disease states. In calcific aortic valve stenosis, Lp(a) is enriched with pro-inflammatory and pro-calcific proteins, supporting disease-specific remodeling of its proteomic cargo [2]. Broader Lp(a) proteome analyses further implicate pathways related to inflammation, coagulation, and tissue repair, highlighting potential mechanistic roles beyond lipid transport [4].

To date, Lp(a) proteomic studies have primarily relied on ultracentrifugation and chromatographic isolation prior to mass spectrometry. However, the overlapping density ranges of Lp(a), LDL, and high-density lipoprotein (HDL), together with limited protein recovery, frequently result in lipoprotein co-purification and proteomic contamination, limiting analytical specificity [5]. In this study we avoid ultracentrifugation and immune-isolate Lp(a) from plasma.

Immunoprecipitation (IP)-based isolation offers a targeted alternative by selectively enriching Lp(a) through apo(a) recognition, thereby increasing confidence that identified proteins are directly associated with Lp(a). Despite this advantage, IP approaches have not been applied to define the Lp(a) proteome [6].

In this study, we compared IP-isolated Lp(a) proteomes generated using in-gel digestion and in-solution proteolysis. We hypothesized that proteins consistently identified across both analytical workflows represent a consistent core Lp(a) proteome and may provide insights into pathways linked to Lp(a)-driven disease.

## 2. Methods

### 2.1. Study Population

The 10 study participants had previously taken part in stable isotope studies of lipoprotein metabolism. Written informed consent was obtained at the time of those studies and included permission for future use of stored samples and associated study data. The proteomic data reported here were generated during method-development work conducted as part of a separate kinetic study in these participants.

Inclusion criteria for the prior studies were that participants (1) were not taking lipid-lowering medications, (2) were not taking over-the-counter supplements, (3) did not have clinical ASCVD, and (4) were in good health based on medical history and physical examination. Subjects were excluded from the study if they (1) had a recent presence (within the last two years) of clinically significant and/or unstable neurological or cardiac disease, (2) were receiving treatment of any kind for hyperlipidemia within 4 weeks of enrollment, or (3) within 30 days of enrollment, used over-the-counter products or consumed food associated with lowering of blood lipids, such as fish oils, flaxseed, red rice, or niacin.

For the present analysis, inclusion criteria were (1) prior consent for future use of stored samples and data and (2) availability of proteomic data generated using both in-solution proteolysis and in-gel digestion.

This study was conducted in accordance with the Declaration of Helsinki, and the protocol was approved by the IRB of Columbia University (IRB-AAAR2511) on 28 November 2017.

In the original cohort, 15 subjects underwent in-solution proteolysis and 17 underwent in-gel digestion; the 10 individuals analyzed by both methods were included in the present study.

### 2.2. Lipid Measurements

Fasting plasma total cholesterol, triglycerides, and HDL cholesterol were measured using an Integra400plus analyzer (Roche, Indianapolis, IN, USA). LDL cholesterol was calculated using the Sampson-NIH equation [7].

### 2.3. Lp(a) Concentration and Apo(a) Isoform Size

Plasma Lp(a) concentrations were quantified using an isoform-independent sandwich ELISA developed and validated by the Northwest Lipid Metabolism and Diabetes Research Laboratory [8]. Apo(a) isoform size was determined by agarose gel electrophoresis as previously described [8,9,10]. Briefly, 250 µL of plasma was diluted in saline to 100 ng of protein in 40 µL, mixed with reducing buffer, electrophoresed overnight at 123 V and 4 °C, transferred to nitrocellulose membranes, immunoblotted, and imaged using the ChemiDoc MP Imaging System (Biorad, Hercules, CA USA). Isoforms were identified by comparison with in-house standards (12–38 KIV2 repeats), and relative expression was quantified using Image Lab software. Intra-sample variability was <15%.

### 2.4. Lp(a) Immunoprecipitation

Lp(a) was isolated from plasma by immunoprecipitation (IP) to enhance specificity for Lp(a)-associated proteins [11]. Dynabeads Protein G (Invitrogen, Cat. No. 10004D, Waltham, MA USA) were incubated with 8 µg of apo(a)-specific rabbit monoclonal antibody (Abcam, Cat. No. ab125014, Waltham, MA USA) for 2 h with rotation at room temperature. The dynabeads were washed with PBS with 0.02% Tween™ 20 (pH 7.4) to remove unbound antibody. Antibody-bound beads were then incubated overnight with rotation at 4 °C with 500 µL of human plasma. After washing, bound Lp(a) was eluted with glycine buffer (pH 2.8) for 1 h at room temperature with rotation and immediately neutralized using 1M Tris (pH 7.5). Eluates were stored at −80 °C until analysis.

### 2.5. Proteolysis and Protein Selection

IP-isolated Lp(a) samples were processed using either in-gel digestion or automated in-solution proteolysis and analyzed by LC–MS/MS. Proteins identified by ≥3 unique peptides and consistently detected across all samples using both methods were included in the final analysis. Detailed descriptions of in-gel and in-solution proteomics methods can be found in the Supplementary Methods, Tables, and Figures. The full proteomics data set can be found in the Supplementary Data—Proteomics Data Excel file.

### 2.6. In-Solution Proteolysis

Automated in-solution proteolysis was performed using the PreON system with iST (96×) columns. Immunoprecipitated Lp(a) samples (~4 µg protein input) were lysed and digested according to the manufacturer’s protocol. Peptides were dried, resuspended in sample loading buffer, and stored at −80 °C. Four to eight technical IP replicates were analyzed per subject.

### 2.7. In-Gel Digestion

Immunoprecipitated samples were separated by 4–12% SDS-PAGE and stained with SimplyBlue. Entire gel lanes were excised and subjected to reduction, alkylation, and overnight trypsin digestion. Peptides were extracted, dried, and resuspended prior to LC–MS/MS analysis [12]. No technical replicates were performed for the in-gel digestion workflow.

## 3. Data Analysis

### 3.1. Lp(a) Protein–Protein Interaction Network and Pathway Analysis

Protein–protein interaction networks and functional enrichment were evaluated using the STRING database (v11.5) [13]. For each method, normalized protein abundances were averaged across subjects and ranked by mean abundance. Networks were constructed using a minimum interaction score of 0.9 (highest confidence). The top 10 enriched Gene Ontology (GO), Biological Processes (BP) and Molecular Functions (MF) were ranked by false discovery rate (FDR). Because STRING enrichment is based on protein identity rather than abundance, GO terms were identical for both methods when restricted to the 34 shared proteins. All statistically significant (*p* < 0.05) biological processes and molecular functions are provided in the Supplementary Data—STRING Networks Excel file in the “All BP” and “All MF” tabs.

### 3.2. Association of the Lp(a) Proteome with Clinical Biomarkers

Associations between protein abundances and clinical biomarkers were assessed using ordinary linear regression for continuous outcomes, including log-transformed Lp(a) concentration, log-transformed HDL cholesterol, log-transformed triglycerides, weighted apo(a) isoform size, total cholesterol, and calculated LDL cholesterol. All models were adjusted for age, sex, and self-reported race and ethnicity.

## 4. Results

### 4.1. Study Population

Ten volunteers, 50% female with a mean age of 47 ± 13.4 years, were analyzed for this study. Median Lp(a) levels were 29.8 nmol/L (IQR 21.7–64.8). Additional population characteristics are listed on Table 1.

### 4.2. Proteome Characterization and Links to Biological Processes

We identified 92 proteins in immunoprecipitated Lp(a) particles using in-solution proteolysis and 55 proteins using in-gel digestion (Supplementary Tables S2 and S3). Thirty-four proteins were shared between methods (Table 2), all of which have been reported previously in independent Lp(a) proteomic studies (Table 3).

### 4.3. Lp(a) Proteome Biological and Molecular Pathway Analysis

**Figure 2:**
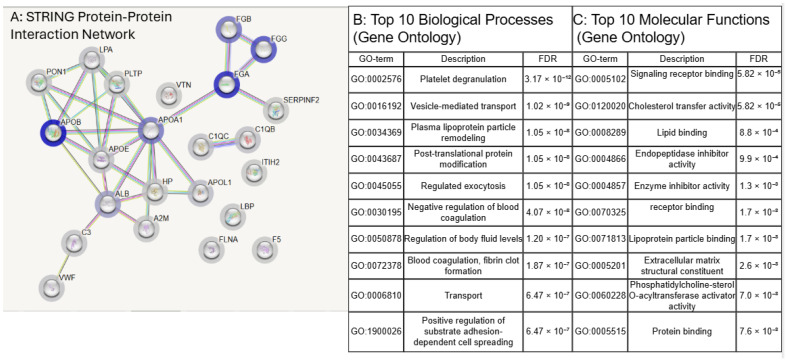
(**A**): STRING protein–protein interaction network of the Lp(a) proteome. The network was generated using STRING 11.5 database, based on the abundances of 34 common proteins identified by in-solution proteolysis preparation. Halo color intensity represents the relative abundance of submitted values. (**B**): Top 10 significantly enriched biological processes based on false discover rate (FDR). (**C**) Top 10 significantly enriched molecular functions based on FDR.

The abundances of the 34 shared proteins were used to construct a working network for isolated Lp(a) particles (Figure 2A) and to identify significantly enriched biological (Figure 2B) and molecular processes (Figure 2C). We concentrated our comparison on these proteins but also provide STRING networks for the 58 proteins unique to in-solution proteolysis and the 21 proteins unique to in-gel digestion (Supplementary Data—STRING Networks, Excel file). Details of those findings can be found in the “Supplemental Lp(a) Proteome Biological and Molecular Pathway Analysis” section of the Supplementary Methods, Tables, and Figures word file.

### 4.4. Association of Proteome with Subject Clinical Biomarkers

Twenty proteins identified by in-solution proteolysis and eleven proteins identified by in-gel digestion were associated with at least one clinical biomarker listed in Table 1 (i.e., Lp(a) concentration, apo(a) isoform size, cholesterol, HDL-C, LDL-C or triglycerides). Notably, there was no overlap between the two datasets (Supplementary Data—Proteomics Data Excel File, “In-Sol Stat Analysis” and “In-Gel Stat Analysis” tabs).

While the overlap of our data with other studies varied, comparison with our unfiltered datasets (i.e., less stringent criteria) revealed substantially greater concordance, indicating that stringent filtering criteria improve specificity but reduce apparent overlaps. These comparisons are summarized in Table 3 and Supplementary Table S4.

## 5. Discussion

The primary objective of this study was to refine characterization of the Lp(a) proteome using IP and to assess the impact of two commonly used proteolysis strategies on proteome definition. By identifying proteins consistently detected across both in-gel and in-solution workflows, we aimed to improve confidence in biologically relevant Lp(a)-associated proteins and the pathways they implicate.

Although in-gel digestion is widely used because of its simplicity, low cost, and ability to remove contaminants, it is known to underrepresent proteins with extreme molecular weights, membrane association, or atypical charge properties. These limitations likely contribute to the proteomic differences observed between in-gel and in-solution methods and underscore the importance of method comparison when defining the Lp(a) proteome [18,19].

To date, seven studies have profiled the Lp(a) proteome—six in human cohorts and one in a transgenic mouse model—reporting between 17 and 208 associated proteins [2,4,14,15,16,17,20]. These prior studies relied on ultracentrifugation-based or chromatography-based isolation, and none employed IP. Human studies uniformly used in-solution digestion, whereas the mouse study used in-gel digestion. Across these investigations, substantial variability in reported proteins highlights the influence of isolation and analytical strategies on proteome composition [2,4,14,15,16,17,20].

Comparison with prior work demonstrates that the 34 proteins identified consistently across both proteolysis methods in our study represent reproducible components of the Lp(a) proteome. Each of these proteins has been detected in at least one previous report and many recur across multiple datasets, supporting their classification as high-confidence Lp(a)-associated proteins. As noted in our results, our stringent filtering criteria improve specificity but reduce apparent overlap between our identified proteins and those present in other published cohorts [2,4,14,15,16,17,20].

Defining biological pathways linked to Lp(a)-associated proteins is critical for understanding how Lp(a) contributes to disease. Although high Lp(a) is an established risk factor for myocardial infarction, stroke, peripheral artery disease, and aortic valve stenosis, the molecular mechanisms driving these associations remain incompletely understood [21]. Functional enrichment of the 34 high-confidence proteins identified previously published and unpublished (exocytosis and endopeptidase activity inhibition—Figure 2) pathways that align with established and emerging roles of Lp(a) in cardiovascular pathology.

Among the most enriched biological processes was plasma lipoprotein particle remodeling, consistent with the formation of Lp(a) through covalent linkage of apo(a) to apoB-100 on LDL particles [22]. Vesicle-mediated transport was also prominent, aligning with evidence that Lp(a) promotes the release of calcifying extracellular vesicles from vascular cells, a key mechanism in aortic valve calcification [17]. Enrichment of negative regulation of coagulation reflects Lp(a)’s structural homology to plasminogen and its capacity to impair fibrinolysis, thereby promoting thrombosis [3]. Additional enrichment of post-translational protein modification is consistent with the pro-inflammatory effects of oxidized phospholipids carried by Lp(a), which can alter protein function and drive atherogenesis [19]. Enriched molecular functions, including lipid binding, protein binding, and endopeptidase inhibitor activity further support these mechanisms [23,24]. Lp(a) is a lipid-rich particle carrying oxidized phospholipids and binds a broad range of extracellular proteins, including fibrinogen, fibronectin, and immunoglobulins, reinforcing roles in extracellular matrix interactions, coagulation, and vascular inflammation [25,26,27].

Importantly, pathway analyses of proteins unique to either proteolysis method revealed limited overlap with pathways identified from the 34 shared proteins, despite partial convergence at broader functional categories. This finding supports the premise that proteins consistently detected across analytical workflows provide more robust biological insight. Similarly, associations between the Lp(a) proteome and clinical biomarkers differed by proteolysis method, highlighting the influence of methodological choice on downstream interpretation.

The current study findings could have clinical implications. First, the identification of immune, inflammatory, and coagulation-related proteins associated with Lp(a) supports the current concept that Lp(a) contributes to ASCVD through multifactorial mechanisms. Enrichment of pathways related to complement activation, fibrinolysis inhibition, and vascular remodeling reinforces the role of Lp(a) as a proinflammatory and prothrombotic mediator, helping to explain its association with myocardial infarction, stroke, and aortic valve disease. Second, the analysis of Lp(a) proteome suggests that cardiovascular risk may not be fully captured by Lp(a) concentration alone, raising the possibility that specific protein components could modify risk and enable more refined, precision-based risk stratification in the future. Third, as Lp(a)-lowering therapies continue to emerge, these data suggest that therapeutic benefits may extend beyond lipid reduction to include modulation of inflammatory and thrombotic pathways, while also highlighting the potential for residual risk driven by non-lipid components. Importantly, the observed variability in protein associations across proteolysis methods underscores the need for methodological standardization before clinical translation of Lp(a) proteomics. Finally, the identification of a consistent core set of Lp(a)-associated proteins supports the concept of Lp(a) as a multifunctional platform particle involved in diverse biological processes, with potential relevance to distinct clinical phenotypes including atherosclerosis and calcific aortic valve disease.

Several limitations warrant consideration. The small sample size limited statistical power and increased the risk of false-negative associations. We did not evaluate the inter-donor variability of the abundances in this study and opted for standardization of abundances as described in supplemental methods. Previous publications have also highlighted that these two methods may pull down different proteins [28,29]. Additionally, the stringent criteria used to define high-confidence proteins may have excluded biologically relevant but less consistently detected proteins. Nevertheless, complementary analyses of method-specific proteins suggest that the core proteome identified here captures the most stable and biologically coherent Lp(a)-associated pathways.

## 6. Conclusions

Using IP and complementary proteolysis strategies, we identified a rigorously defined core Lp(a) proteome comprising 34 consistently detected proteins. Network and functional analyses implicate pathways related to lipoprotein remodeling, vesicle-mediated transport, coagulation regulation, and protein binding, providing mechanistic insight into how Lp(a) may promote vascular inflammation, thrombosis, and calcification. Together, these findings refine the molecular framework of the Lp(a) proteome and highlight the importance of methodological rigor in proteomic studies. Future investigations in larger cohorts, coupled with functional validation, are needed to determine how variation in the Lp(a) proteome contributes to disease risk and to identify potential therapeutic targets.

## Figures and Tables

**Table 1:** Baseline characteristics.

Subject	Gender	Race	Age	Lp(a) Level nmol/L	Apo(a) Isoform 1	% Isoform 1	Apo(a) Isoform 2	% Isoform 2	Cholesterol mg/dL	HDL mg/dL	LDL-C mg/dL	Triglycerides mg/dL
1	Female	White	29	15	20	84%	27	16%	173	62	103	40
2	Male	Hispanic	44	21	24	88%	17	12%	138	33	88	90
3	Female	Hispanic	60	22	21	60%	34	40%	192	44	126	120
4	Female	White	62	22	26	100%	-	-	160	54	84	122
5	Female	Hispanic	66	23	14	61%	28	39%	183	39	111	190
6	Female	Black	31	37	19	50%	26	50%	232	44	176	71
7	Male	Black	28	57	26	94%	22	6%	124	53	56	72
8	Male	Black	50	67	18	50%	19	50%	156	70	72	73
9	Male	Black	57	89	21	97%	30	3%	139	60	68	57
10	Male	Black	47	146	22	79%	26	21%	177	33	116	153
Median (IQR)									167 (143,182)	49 (40,58)	94 (74,110)	82 (71,110)

**Table 2:** Ranking 34 proteins common between in-solution proteolysis and in-gel digestion based on in-solution abundances.

Rank	Protein Name	Gene Name	Atherosclerosis	Thrombosis	Inflammation
1	Immunoglobulin heavy constant gamma 1 (Fragment)	IGHG1	–	–	✓
2	Apolipoprotein B-100	APOB	✓	–	✓
3	Fibrinogen alpha chain	FGA	✓	✓	✓
4	Immunoglobulin kappa constant	IGKC	–	–	✓
5	Fibrinogen gamma chain	FGG	✓	✓	✓
6	Fibrinogen beta chain	FGB	✓	✓	✓
7	Apolipoprotein A-I	APOA1	✓ *	–	✓ *
8	Serum Albumin	ALB	–	–	✓ (indirect)
9	Immunoglobulin heavy constant gamma 2 (Fragment)	IGHG2	–	–	✓
10	Immunoglobulin heavy constant mu	IGHM	✓ (immune-related)	–	✓
11	Immunoglobulin heavy constant gamma 3 (Fragment)	IGHG3	–	–	✓
12	Apolipoprotein L1	APOL1	–	–	✓
13	Complement C1q subunit B	C1QB	✓	✓	✓
14	Apolipoprotein(a)	LPA	✓	✓	✓
15	Immunoglobulin heavy variable 3-72	IGHV3-72	–	–	✓
16	von Willebrand factor	VWF	✓	✓	✓
17	Complement C1q subunit C	C1QC	✓	✓	✓
18	Apolipoprotein E	APOE	✓	–	✓
19	Immunoglobulin heavy constant alpha 1	IGHA1	–	–	✓
20	Immunoglobulin heavy variable 6-1	IGHV6-1	–	–	✓
21	Immunoglobulin heavy constant gamma 4	IGHG4	–	–	✓
22	Complement C3	C3	✓	✓	✓
23	Haptoglobin	HP	✓	–	✓
24	Filamin-A	FLNA	(limited)	(limited)	(limited)
25	Lipopolysaccharide-binding protein	LBP	✓	–	✓
26	Paraoxonase 1	PON1	✓ *	–	✓ *
27	Alpha-2-macroglobulin	A2M	–	✓	✓
28	Immunoglobulin heavy variable 2-70D	IGHV2-70D	–	–	✓
29	Immunoglobulin heavy variable 3-49	IGHV3-49	–	–	✓
30	Inter-alpha-trypsin inhibitor heavy chain H2	ITIH2	–	–	✓
31	Coagulation factor V	F5	–	✓	✓
32	Phospholipid transfer protein	PLTP	✓	–	✓
33	Vitronectin	VTN	✓	✓	✓
34	Alpha-2-antiplasmin	SERPINF2	–	✓	–

✓ indicates prior literature linking the protein to atherosclerosis, thrombosis, and/or inflammation (PubMed search). “–” indicates that a link was not found in the literature. For some proteins (particularly immunoglobulins), associations reflect broader immune and inflammatory processes rather than direct causal roles in vascular disease. Asterisks (*) denote proteins with generally protective or anti-atherogenic functions.

**Table 3:** Comparison of the previous literature—proteins in common with 34 identified proteins.

Publication	Methods	Gene Name of Proteins in Common
Von Zychlinski et al. 2011 [4]	Ultracentrifugation, size exclusion chromatography, 2D nano LC-MS/MS, AQUA peptide quantification	APOB, C3, LPA, ALB, APOA1, APOE, FGB, A2M, FGG, FGA, LBP, VTN, IGHG1, APOL1, PON1 (*n* = 15)
Von Zychlinski et al. 2014 [14]	Ultracentrifugation + FPLC, ^31^P NMR spectroscopy, nano LC-MS/MS	APOB, LPA, APOA1, APOE, C3, PON1 (*n* = 6)
Bourgeois et al. 2021 [2]	Ultracentrifugation, Label-free nano LC-MS/MS, Transcriptomics	APOB, LPA, APOA1, APOE, HP, PON1, IGHA1, C3, IGKC, VTN, ITIH2, IGHM, IGHG1, F5, FLNA, A2M, PLTP, LBP, IGHG2, SERPINF2, APOL1, IGHG3 (*n* = 22)
Bourgeois et al. 2021 [15]	Ultracentrifugation + FPLC, nano LC-MS/MS, PRM, MR	ITIH2, VTN, PON1 (*n* = 3)
Mueller et al. 2022 [16]	Ultracentrifugation + FPLC, LC–MS/MS proteomics, NTA, immunoblotting	APOB, LPA, APOA1, APOE, F5, FGG, IGHA1, FGB, PON1, VTN, APOL1, IGHG1, C3 (*n* = 13)
Rogers et al. 2022 [17]	Ultracentrifugation, size exclusion chromatography, single-vesicle flow cytometry, STORM super-resolution microscopy, LC–MS/MS proteomics	A2M, ALB, APOA1, APOB, APOE, APOL1, C1QB, C1QC, C3, F5, FGA, FGG, FLNA, HP, IGHA1, IGHG1, IGHG2, IGHG3, IGHG4, IGHM, IGHV2-70D, IGHV3-49, IGHV3-72, IGHV6-1, IGKC, ITIH2, LBP, LPA, PLTP, PON1, SERPINF2, VTN, VWF (*n* = 33)

## Data Availability

The original contributions presented in this study are included in the article/Supplementary Material. Further inquiries can be directed to the corresponding author(s).

## Supplementary Materials

The following supporting information can be downloaded at: https://www.mdpi.com/article/10.3390/jcm15072559/s1, Method S1: In-Solution Proteolysis Method, Method S2: In-Gel Digestion Method, Table S1: Ranking 34 Proteins Common Between In-Solution Proteolysis and In-Gel Digestion Based In-Gel Abundances, Table S2: 92 Proteins Identified By In-Solution Proteolysis, Table S3: 55 Proteins Identified By In-Gel Digestion, Table S4: Comparison to Previous Literature, Figure S1: In-Gel Digestion STRING Network, Analysis S1: Lp(a) Proteome Biological and Molecular Pathway Analysis. All Proteomics Data can be found in the Supplementary Data—Proteomics Data Excel file. All STRING Networks can be found in the Supplementary Data—STRING Networks Excel file [30].
