# Supplementary material for: Comparative Proteomic Analysis of Lipoprotein(a): Method-Dependent Profiles and Disease Pathways
Source: J Clin Med. 2026 Mar 27;15(7):2559. doi: 10.3390/jcm15072559 (PMC13073900; doi:10.3390/jcm15072559)
Supplement: Supplementary file 1 [file jcm-15-02559-s001.zip › Supplementary Methods, Tables, and Figures.pdf]

Comparative Proteomic Analysis of Lipoprotein(a): Method-Dependent Profiles and Disease Pathways

Nelsa Matienzo [1,†], Zoe Kress [1,†], Sasha A. Singh [2], Masanori Aikawa [2,3,4], Rajesh K. Soni [5], Yihao Li [1] and Gissette Reyes-Soffer [1,*]

1 Division of Preventive Medicine and Nutrition, Department of Medicine, Columbia University Vagelos College of Physicians and Surgeons, 630 West 168th street, P&S 8-503, New York, NY 10032, USA
2 Center for Interdisciplinary Cardiovascular Sciences, Division of Cardiovascular Medicine, Department of Medicine, Brigham Women's Hospital, Harvard Medical School, Boston, MA 02115, USA
3 Center for Excellence in Vascular Biology, Division of Cardiovascular Medicine, Brigham and Women's Hospital, Harvard Medical School, Boston, MA 02115, USA
4 Channing Division of Network Medicine, Department of Medicine, Brigham Women's Hospital, Harvard Medical School, Boston, MA 02115, USA
5 Proteomics and Macromolecular Crystallography Shared Resource, Herbert Irving Comprehensive Cancer Center, Columbia University, New York, NY 10032, USA;
* Correspondence: gr2104@cumc.columbia.edu
† These authors contributed equally to this work.

Supplementary Methods

Method S1: In-Solution Proteolysis Method

Immunoprecipitated (IP) Lp(a) particle in-solution proteolysis.

We used automated proteolysis, using the PreON (PreOmics GmbH, Germany) with the iST (96x) columns (PreOmics). 40 ul of the iST LYSE buffer is added to 10 ul IPed Lp(a) sample, corresponding to an average 4 ug protein input and subsequent proteolysis steps are done on the PreON (12-24 samples a day). Eluted peptides are dried in a speed vacuum and then re-suspended in 5% acetonitrile and 0.5% formic acid dissolved in MS-grade water (sample loading buffer) and stored at -80°C.

Data-dependent acquisitions (DDA) of the LPA proteome.

The peptides (2:1 dilution as above, 2 ul injected) were analyzed with the following settings: the Lumos is set to 120 K resolution, and the top N precursor ions in a 3 second cycle time (within a scan range of 375-1500 m/z) is subjected to higher energy dissociation (HCD, collision energy 30%) for peptide sequencing using a 30 K resolution setting. The parallelization feature is enabled (automatic gain control target, 1.0e5; maximum injection time, 54 ms). The gradient flow rate was 300 nl/min from 8 to 25% solvent B (acetonitrile/0.1% formic acid) for 30 minutes, 25 to 30% Solvent B for 10 minutes, followed by an additional 4 minutes of 95 % solvent B.

Lp(a) Proteome Profiling

The Lp(a) proteome was exported to generate a single protein abundance value per subject. Proteins with 3 or more unique peptides and present in each subject were considered further.

Method S2: In-Gel Digestion Method

Immunoprecipitated samples were separated on 4–12% gradient SDS-PAGE gels and stained with SimplyBlue (Thermo Fisher Scientific). Protein bands were excised, and in-gel digestion was performed as described previously (Shevchenko et al., 2006), with minor modifications. Gel slices were washed with 1:1 (acetonitrile: 100 mM ammonium bicarbonate) for 30 min, dehydrated with 100% acetonitrile for 10 min until shrunken, and dried in a SpeedVac for 10 min without heat. Reduction was performed with 5 mM DTT for 30 min at 56 °C in an air thermostat, followed by cooling to room temperature. Alkylation was carried out with 11 mM IAA for 30 min in the dark. Gel slices were sequentially washed with 100 mM ammonium bicarbonate and 100% acetonitrile for 10 min each, dried in a SpeedVac, and rehydrated in 25 ng/μL trypsin in 50 mM ammonium bicarbonate on ice for 30 min. Digestion was performed overnight at 37 °C in an air thermostat. Peptides were extracted from the gel slices with extraction buffer (1:2, v/v, 5% formic acid/acetonitrile) under high-speed shaking. Supernatants from the digestion and extraction steps were combined and dried in a SpeedVac. Peptides were resuspended in 3% acetonitrile/0.1% formic acid prior to LC–MS/MS analysis.

LC–MS/MS analysis

Peptide separation was performed using a Thermo Scientific™ UltiMate™ 3000 RSLCnano system with a Thermo Scientific EASY-Spray™ source. Samples were first loaded onto a Thermo Scientific™ Acclaim™ PepMap™ 100 trap column (2 cm × 75 μm) and then separated on a Thermo Scientific™ EASY-Spray™ PepMap™ RSLC C18 analytical column (50 cm × 75 μm ID) using a 5–45% acetonitrile gradient in 0.1% formic acid over 100 min at a flow rate of 250 nL/min. The column temperature was maintained at 50 °C. Mass spectrometric analysis was performed on a Thermo Scientific™ Orbitrap Fusion™ Tribrid™ mass spectrometer. Survey scans of peptide precursors were acquired from 350–1650 m/z at 120,000 FWHM resolution (at 200 m/z) with an AGC target of 3 × 106 and a maximum injection time of 50 ms. The instrument was operated in top-speed mode with 3 s cycles for MS and MS/MS scans. Precursors with charge states 2–6 and intensities above 3 × 104 were selected for fragmentation by quadrupole isolation at 1.4 Th, followed by CID at 28% collision energy. Fragments were detected in the ion trap at rapid scan rate with an AGC target of 1 × 105 and a maximum injection time of 100 ms. Dynamic exclusion was set to 45 s with a 10-ppm mass tolerance. Monoisotopic precursor selection was enabled.

Data analysis

Raw MS data were processed in MaxQuant (v1.6.1.0) (Cox and Mann, 2008) using the Andromeda search engine (Cox et al., 2011). The first search and main search tolerances

were set to 20 ppm and 6 ppm, respectively. The reference human proteome database was downloaded from UniProt. Trypsin specificity was set with up to two missed cleavages. Peptide, site, and protein false discovery rates (FDR) were set to 1%, with a minimum of one peptide required for identification. Label-free quantification (LFQ) was enabled with a minimum ratio of 1. Variable modifications included oxidation (M), protein N-terminal acetylation, and deamidation (N/Q).

References:

1. Shevchenko A, Tomas H, Havlis J, Olsen JV, Mann M In-gel digestion for mass spectrometric characterization of proteins and proteomes. Nat Protoc.2006;1(6):2856-60.

2. Cox, J., and Mann, M. (2008). MaxQuant enables high peptide identification rates, individualized p.p.b.-range mass accuracies and proteome-wide protein quantification. Nat. Biotechnol. 26, 1367–1372.

3. Cox, J., Neuhauser, N., Michalski, A., Scheltema, R.A., Olsen, J.V., and Mann, M. (2011). Andromeda: a peptide search engine integrated into the MaxQuant environment. J. Proteome Res. 10, 1794–1805

Supplementary Tables

Table S1: Ranking 34 Proteins Common Between In-Solution Proteolysis and In-Gel Digestion Based In-Gel Abundances

Rank	Protein Name	Gene Name
**1**	Immunoglobulin heavy constant gamma 1 (Fragment)	IGHG1
**2**	Fibrinogen alpha chain	FGA
**3**	Fibrinogen gamma chain	FGG
**4**	Fibrinogen beta chain	FGB
**5**	Apolipoprotein A-I	APOA1
**6**	Apolipoprotein B-100	APOB
**7**	Immunoglobulin heavy constant gamma 3 (Fragment)	IGHG3
**8**	Immunoglobulin heavy constant gamma 2 (Fragment)	IGHG2
**9**	Apolipoprotein(a)	LPA
**10**	Isoform 2 of Immunoglobulin heavy constant mu	IGHM
**11**	Immunoglobulin kappa constant	IGKC
**12**	Serum Albumin	ALB
**13**	von Willebrand factor	VWF
**14**	Immunoglobulin heavy constant gamma 4 (Fragment)	IGHG4
**15**	Complement C1q subcomponent subunit B	C1QB
**16**	Complement C3	C3
**17**	Immunoglobulin heavy constant alpha 1 (Fragment)	IGHA1
**18**	Apolipoprotein E	APOE

**19**	Immunoglobulin heavy variable 6-1	IGHV6-1
**20**	Lipopolysaccharide-binding protein	LBP
**21**	Alpha-2-macroglobulin	A2M
**22**	Vitronectin	VTN
**23**	Haptoglobin	HP
**24**	Isoform 2 of Apolipoprotein L1	APOL1
**25**	Immunoglobulin heavy variable 3-49	IGHV3-49
**26**	Filamin-A	FLNA
**27**	Coagulation factor V	F5
**28**	Complement C1q subcomponent subunit C	C1QC
**29**	Serum paraoxonase/arylesterase 1	PON1
**30**	Immunoglobulin heavy variable 2-70D	IGHV2-70D
**31**	Immunoglobulin heavy variable 3-72	IGHV3-72
**32**	Inter-alpha-trypsin inhibitor heavy chain H2	ITIH2
**33**	Alpha-2-antiplasmin	SERPINF2
**34**	Phospholipid transfer protein	PLTP

Table S2: 92 Proteins Identified By In-Solution Proteolysis		
**Rank**	**Protein Name**	**Gene Name**
**1**	Immunoglobulin heavy constant gamma 1 (Fragment)	IGHG1
**2**	Apolipoprotein B-100	APOB
**3**	Fibrinogen alpha chain	FGA
**4**	Immunoglobulin kappa constant	IGKC
**5**	Immunoglobulin lambda constant 2	IGLC2
**6**	Fibrinogen gamma chain	FGG
**7**	Fibrinogen beta chain	FGB
**8**	Apolipoprotein A-I	APOA1
**9**	Albumin	ALB
**10**	Immunoglobulin heavy constant gamma 2 (Fragment)	IGHG2
**11**	Isoform 1 of Fibronectin	FN1
**12**	Isoform 2 of Immunoglobulin heavy constant mu	IGHM
**13**	Immunoglobulin heavy constant gamma 3 (Fragment)	IGHG3
**14**	Hemoglobin subunit beta	HBB
**15**	Isoform 2 of Clusterin	CLU
**16**	Isoform 2 of Apolipoprotein L1	APOL1
**17**	Complement C1q subcomponent subunit B	C1QB
**18**	Hemoglobin subunit alpha	HBA1
**19**	Apolipoprotein(a)	LPA
**20**	Immunoglobulin heavy variable 3-72	IGHV3-72
**21**	Apolipoprotein C-III	APOC3
**22**	Immunoglobulin kappa variable 1-33	IGKV1D-33
**23**	von Willebrand factor	VWF
**24**	Complement C1q subcomponent subunit C	C1QC
**25**	Apolipoprotein E	APOE
**26**	Immunoglobulin heavy constant alpha 1 (Fragment)	IGHA1
**27**	Isoform 3 of Vitamin D-binding protein	GC
**28**	Immunoglobulin heavy variable 6-1	IGHV6-1
**29**	Myosin regulatory light chain 2, ventricular/cardiac muscle isoform	MYL2
**30**	Immunoglobulin heavy constant gamma 4 (Fragment)	IGHG4
**31**	Complement C3	C3
**32**	Haptoglobin	HP
**33**	Myosin-9	MYH9
**34**	Filamin-A	FLNA

**35**	Apolipoprotein C-I	APOC1
**36**	Lipopolysaccharide-binding protein	LBP
**37**	Isoform 2 of Heat shock protein HSP 90-alpha	HSP90AA1
**38**	Serum paraoxonase/arylesterase 1	PON1
**39**	Immunoglobulin J chain	JCHAIN
**40**	Actin, cytoplasmic 1	ACTB
**41**	Heparin cofactor 2	SERPIND1
**42**	Vimentin	VIM
**43**	Complement C4-B	C4B
**44**	Ig-like domain-containing protein (Fragment)	Not Available
**45**	Alpha-2-macroglobulin	A2M
**46**	Serotransferrin	TF
**47**	Apolipoprotein C-II	APOC4-APOC2
**48**	Immunoglobulin heavy variable 2-70D	IGHV2-70D
**49**	Coagulation factor XIII A chain	F13A1
**50**	Gelsolin	GSN
**51**	Apolipoprotein A-IV	APOA4
**52**	Complement component C9	C9
**53**	Thrombospondin-1	THBS1
**54**	Isoform 4 of Collagen alpha-3(VI) chain	COL6A3
**55**	Sulfhydryl oxidase 1	QSOX1
**56**	Talin-1	TLN1
**57**	Immunoglobulin heavy variable 3-49	IGHV3-49
**58**	Apolipoprotein D (Fragment)	APOD
**59**	Inter-alpha-trypsin inhibitor heavy chain H2	ITIH2
**60**	Coagulation factor V	F5
**61**	Alpha-1-antichymotrypsin	SERPINA3
**62**	Cholesteryl ester transfer protein	CETP
**63**	Inter-alpha-trypsin inhibitor heavy chain H1	ITIH1
**64**	ITIH4 protein	ITIH4
**65**	Peroxiredoxin-2	PRDX2
**66**	Isoform 3 of Alpha-actinin-1	ACTN1
**67**	Isoform 2 of Haptoglobin-related protein	HPR
**68**	Phospholipid transfer protein	PLTP
**69**	Complement C1s subcomponent	C1S
**70**	Elongation factor 1-alpha 1	EEF1A1
**71**	SAA2-SAA4 readthrough	SAA2-SAA4
**72**	Alpha-1-acid glycoprotein 2	ORM2

**73**	Immunoglobulin lambda variable 3-10	IGLV3-10
**74**	Transitional endoplasmic reticulum ATPase	VCP
**75**	Alpha-2-HS-glycoprotein	AHSG
**76**	Desmoplakin	DSP
**77**	Transthyretin	TTR
**78**	Glyceraldehyde-3-phosphate dehydrogenase	GAPDH
**79**	Vitronectin	VTN
**80**	Collagen alpha-1(XIV) chain	COL14A1
**81**	Alpha-2-antiplasmin	SERPINF2
**82**	Alpha-1-acid glycoprotein 1	ORM1
**83**	Antithrombin-III	SERPINC1
**84**	Kininogen-1	KNG1
**85**	Integrin alpha-IIb	ITGA2B
**86**	Cofilin, non-muscle isoform	CFL1
**87**	Prenylcysteine oxidase 1	PCYOX1
**88**	Retinal dehydrogenase 1	ALDH1A1
**89**	Histidine-rich glycoprotein	HRG
**90**	Angiotensinogen	AGT
**91**	Prothrombin	F2
**92**	Selenoprotein P	SELENOP

Table S3: 55 Proteins Identified By In-Gel Digestion		
**Rank**	**Protein Name**	**Gene Name**
**1**	Ig gamma-1 chain C region	IGHG1
**2**	Fibrinogen alpha chain	FGA
**3**	Fibrinogen gamma chain	FGG
**4**	Fibrinogen beta chain	FGB
**5**	Apolipoprotein A-I	APOA1
**6**	Apolipoprotein B-100	APOB
**7**	Ig gamma-3 chain C region	IGHG3
**8**	Ig gamma-2 chain C region	IGHG2
**9**	Apolipoprotein(a)	LPA
**10**	Ig mu chain C region	IGHM
**11**	Ig kappa chain C region	IGKC
**12**	Complement C1q subcomponent subunit A	C1QA
**13**	Serum albumin	ALB
**14**	von Willebrand factor	VWF
**15**	Ig kappa chain V-III region B6	IGKV3
**16**	Ig gamma-4 chain C region	IGHG4
**17**	Complement C1q subcomponent subunit B	C1QB
**18**	Immunoglobulin lambda-like polypeptide 5	IGLL5;IGLC1
**19**	Complement C3	C3
**20**	Ig alpha-1 chain C region	IGHA1
**21**	Apolipoprotein E	APOE
**22**	CD5 antigen-like	CD5L
**23**	Immunoglobulin heavy variable 6-1	IGHV6-1
**24**	Ig heavy chain V-I region V35	IGHV1-2
**25**	Ig kappa chain V-IV region	IGKV4-1
**26**	Lipopolysaccharide-binding protein	LBP
**27**	Alpha-2-macroglobulin	A2M
**28**	Complement C4-A	C4A
**29**	Vitronectin	VTN
**30**	Immunoglobulin heavy variable 3-15	IGHV3-15
**31**	Ig kappa chain V-I region HK102	IGKV1-5
**32**	Haptoglobin	HP
**33**	Alpha-1-antitrypsin;Short peptide from AAT	SERPINA1
**34**	Immunoglobulin heavy variable 4-28	IGHV4-28
**35**	Apolipoprotein L1	APOL1
**36**	Immunoglobulin heavy variable 3-49	IGHV3-49
**37**	Filamin-A	FLNA
**38**	Ig lambda chain V-III region SH	IGLV3-19
**39**	Coagulation factor V	F5
**40**	Complement C1q subcomponent subunit C	C1QC

**41**	Immunoglobulin heavy variable 5-51	IGHV5-51
**42**	Serum amyloid A-4 protein	SAA4
**43**	Serum paraoxonase/arylesterase 1	PON1
**44**	Immunoglobulin kappa variable 2-24	IGKV2-24;IGKV2D-24
**45**	Immunoglobulin heavy variable 2-70D	IGHV2-70D
**46**	Immunoglobulin heavy variable 3-72	IGHV3-72
**47**	Ig kappa chain V-III region VG	IGKV3D-11
**48**	Beta-2-glycoprotein 1	APOH
**49**	Inter-alpha-trypsin inhibitor heavy chain H2	ITIH2
**50**	Ig kappa chain V-II region RPMI 6410	IGKV2D-30
**51**	Ig kappa chain V-I region BAN	IGKV1-16
**52**	Alpha-2-antiplasmin	SERPINF2
**53**	Complement C1r subcomponent	C1R
**54**	Phospholipid transfer protein	PLTP
**55**	Fermitin family homolog 3	FERMT3

Table S4 can be found in the Excel file named "Supplementary Table 4 - Comparison to Previous Literature"

Supplementary Figures

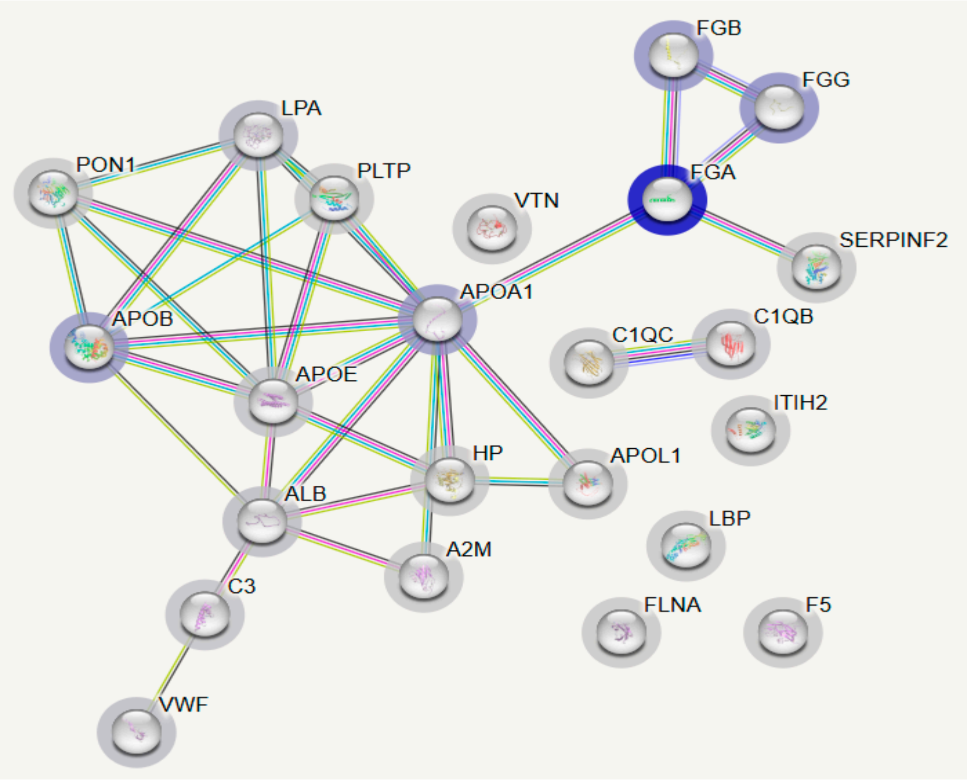

Figure S1: In-Gel Digestion STRING Network Lp(a) proteome biological network generated using the STRING 11.5 database based on the abundances of the 34 common proteins according to the in-gel digestion preparation. Halo color intensity is based on abundance values submitted.

Supplementary Analysis

Analysis S1: Lp(a) Proteome Biological and Molecular Pathway Analysis

Among the Top 10 Biological Processes for the 34 shared proteins, four overlapped with the 58 in-solution–specific proteins and one overlapped with the 21 in-gel–specific proteins. For the Top 10 Molecular Functions, four overlapped with the in-solution–specific proteins, while there were insufficient in-gel–specific proteins to generate a comparable table. Across all identified processes, 75 of 198 Biological Processes for the in-solution–specific proteins and 10 of 14 for the in-gel–specific proteins overlapped with the 168 Biological Processes identified from the 34 shared proteins. Similarly, 12 of 33 Molecular Processes for the in-solution–specific proteins overlapped with the 19 Molecular Processes from the shared proteins, with insufficient in-gel–specific proteins to generate molecular functions. These analyses are summarized in the Supplementary Data (STRING Networks Excel File, "Top 10 BP Venn Diagram," "Top 10 MF Venn Diagram," "All BP Venn Diagram," and "All MF Venn Diagram" tabs).
